# Bioenergy and African transformation

**DOI:** 10.1186/s13068-014-0188-5

**Published:** 2015-02-12

**Authors:** Lee R Lynd, Mariam Sow, Annie FA Chimphango, Luis AB Cortez, Carlos H Brito Cruz, Mosad Elmissiry, Mark Laser, Ibrahim A Mayaki, Marcia AFD Moraes, Luiz AH Nogueira, Gideon M Wolfaardt, Jeremy Woods, Willem H van Zyl

**Affiliations:** Thayer School of Engineering, Dartmouth College, Hanover, NH USA; New Partnership for Africa’s Development (NEPAD), Johannesburg, South Africa; Department of Process Engineering, University of Stellenbosch, Stellenbosch, South Africa; Faculty of Agricultural Engineering, University of Campinas, Campinas, Brazil; São Paulo Research Foundation São Paulo, São Paulo, Brazil; Physics Institute, University of Campinas, Campinas, Brazil; Department of Applied Economics, University of São Paulo, ESALQ, Piracicaba, Brazil; Department of Microbiology, University of Stellenbosch, Stellenbosch, South Africa; Water Institute, University of Stellenbosch, Stellenbosch, South Africa; Centre for Environmental Policy, Imperial College London, London, UK

## Abstract

Among the world’s continents, Africa has the highest incidence of food insecurity and poverty and the highest rates of population growth. Yet Africa also has the most arable land, the lowest crop yields, and by far the most plentiful land resources relative to energy demand. It is thus of interest to examine the potential of expanded modern bioenergy production in Africa. Here we consider bioenergy as an enabler for development, and provide an overview of modern bioenergy technologies with a comment on application in an Africa context. Experience with bioenergy in Africa offers evidence of social benefits and also some important lessons. In Brazil, social development, agricultural development and food security, and bioenergy development have been synergistic rather than antagonistic. Realizing similar success in African countries will require clear vision, good governance, and adaptation of technologies, knowledge, and business models to myriad local circumstances. Strategies for integrated production of food crops, livestock, and bioenergy are potentially attractive and offer an alternative to an agricultural model featuring specialized land use. If done thoughtfully, there is considerable evidence that food security and economic development in Africa can be addressed more effectively with modern bioenergy than without it. Modern bioenergy can be an agent of African transformation, with potential social benefits accruing to multiple sectors and extending well beyond energy supply per se. Potential negative impacts also cut across sectors. Thus, institutionally inclusive multi-sector legislative structures will be more effective at maximizing the social benefits of bioenergy compared to institutionally exclusive, single-sector structures.

## Introduction

Universal access to affordable, reliable, and modern energy services is and will increasingly be required for growth and development across Africa. As such, energy provision will be a central pillar in national and regional industrialization policy and strategies. In turn, delivering energy services is a critical component of the advancement of agriculture as a basis for a broad and inclusive socio-economic growth and development strategy. In this regard, bioenergy is already playing a central role in food production and provision and is considered in most developed countries as one among several routes for diversification of energy sources. Its role might be more crucial in Sub-Saharan Africa, where so many are entirely dependent on access to land and its products, which include traditional forms of bioenergy, to survive.

With annual gross domestic product (GDP) growth rates reaching 5% during the past decade, more than twice that of the 1980s and 1990s, Africa has become one of the fastest growing continents. However, this growth has not been equally distributed and, despite substantial progress made in creating skills and jobs, poverty and food insecurity are still widespread. According to the most recent estimates available, 47% of the population of Sub-Saharan Africa lives on less than $1.25 per day, and 27% are hungry or undernourished [1]. 43% of Africans have no access to electricity, and this percentage rises to 80% in rural areas [2]. The situation in some African countries is much worse. The challenge of addressing these issues is further heightened by population demographics featuring two-thirds of the population below 25 years of age, most of whom are unemployed. According to the UN Population Division, “the largest regional percentage increase in population between 2013 and 2050 will be in Africa, whose population can be expected to at least double and increase from 1.3 billion to about 2.3 billion, with a further 1.8 billion increase between 2050 and 2100. That projection, however, depends on the assumption that sub-Saharan Africa’s total fertility rate (the average number of children per woman) will decline from 5.1 to approximately 3.0 by 2050” [3], which is yet to be supported by data.

The New Partnership for Africa’s Development (NEPAD) Agency, along with regional organizations, believes that innovative approaches beyond business-as-usual should be undertaken to address Africa’s multiple, interconnected challenges. Such approaches are adopted through the transformation agenda designed and implemented by the continental and regional bodies, and include among others: 1) the Comprehensive Africa Agriculture Development Programme (CAADP) Framework, 2) the Program for Infrastructure Development in Africa (PIDA), and more recently 3) the Rural Futures Program [4]. These programs are about fostering transformation. Such a transformation has been defined as “a people-centered approach based on equity and inclusiveness where rural men and women can develop their potential and reach their aspirations including income security, whilst securing environmental sustainability and where all territories in a country can express their development potential and none of them are persistently marginalized” [4]. This innovative approach is based on three basic principles: economic profitability, social equity, and environmental sustainability. Well-designed and implemented bioenergy strategies can contribute substantially to this transformation goal. In particular, modern bioenergy brings a distinctive set of attributes such that the range of development approaches and outcomes with bioenergy is substantially expanded, and can in some cases be improved, as compared to the case without bioenergy.

**Figure 1:**
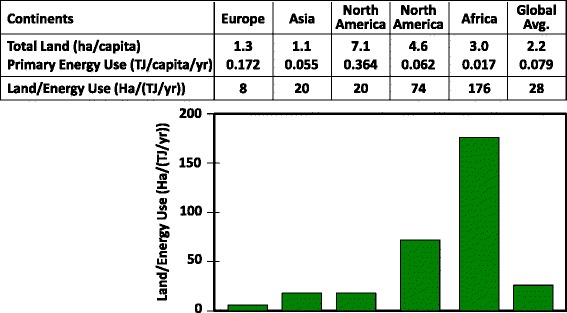
**Comparative land resources and energy demand.** Sources: population [6]; energy [7]; land [8].

In considering the many intricacies and challenges associated with bioenergy and development in Africa, it is important to not lose sight of the obvious: bioenergy provides a route for Africans, from the most vulnerable to the wealthiest, to obtain critically needed energy from a resource in which the continent is rich, that is, land. To equal the land area of Africa, one can add that of China, India, Europe, and the United States - which together represent just under half the world’s population. Africa has the most arable land of any continent, a substantial fraction of land well suited for production of rain-fed crops that is not currently cultivated, and the lowest per hectare crop yields in the world [5]. The potential to increase the production and harvest of biomass for both food and energy is thus very large. With land per capita above the global average and by far the lowest per capita primary energy use in the world, Africa’s land resources are uniquely plentiful relative to demand for energy (Figure 1). Africa’s singularly high ratio of bioenergy potential compared to current demand may of course change somewhat in the course of future development, and this will be important to consider.

Translating this potential into reality requires that daunting challenges be overcome, including those that have limited development in the agricultural sector for decades, such as widespread lack of agricultural extension, degraded soils, poorly developed infrastructure, conflict and poor governance, and complications associated with land tenure. Also critical is the availability of water resources and competing demands for land use including food and fiber crops, pasture, timber, and the whole range of forest products which constitute a substantial component of local populations’ food security and well-being in terms of health. Ultimately, bioenergy cannot solve Africa’s longstanding problems by itself and must be seen as one tool among many in the context of a systemic approach.

Bioenergy production requires land, and is thus inextricably linked with social development, agriculture, and environmental quality. These linkages increase the complexity of analysis and deployment of bioenergy, and can result in undesirable consequences if managed poorly. If managed well, they also have potential to greatly multiply the benefits beyond energy provision per se. Illustrative of the potential for bioenergy to be a double-edged sword, a 2011 working paper prepared by Practical Action Consulting [9] observes that biofuels development has the potential to produce a paradigm shift in agriculture, industrial, and rural development in Africa, while simultaneously providing opportunities to significantly increase energy self-sufficiency. However, the working paper also notes that ineffective policies risk displacing indigenous populations, agricultural productivity, and ecosystems for crops that may, in some cases, fail.

There is thus both a moral imperative to consider and beneficially deploy bioenergy to address critical issues on the African continent at the same time that there is potential to deploy bioenergy in harmful ways. Clear vision, strong policies, and good governance will likely be essential in order for the potential of bioenergy to be realized, and they represent an urgent need. Our objective in writing this paper is to be responsive to this need.

## Bioenergy as a potential enabler of development

As noted by the United Nations Development Program (sustainable energy) “Energy is central to sustainable development and poverty reduction efforts. It affects all aspects of development -- social, economic, and environmental -- including livelihoods, access to water, agricultural productivity, health, population levels, education, and gender-related issues. None of the Millennium Development Goals (MDGs) can be met without major improvement in the quality and quantity of energy services in developing countries” [10].

Several potential contributions of bioenergy to development are listed by Lynd and Woods [11], including employment; development of marketable and transferable skills for the rural population; introduction of agricultural infrastructure and know-how; improved balance of payments and currency valuation; energy democratization, self-sufficiency, and availability for agricultural machinery and processing; and an economically rewarding way to regenerate Africa’s vast areas of degraded land. A substantial literature points to disproportionately large benefits to the rural poor from agricultural development as compared to other kinds of development [12-14].

A comprehensive study of 15 small-scale bioenergy projects in 12 countries, 5 from Africa [15], drew preliminary lessons and conclusions as follows:

- Natural resource efficiency is possible in small-scale bioenergy initiatives.
- Local and productive energy end uses develop virtuous circles.
- Where fossil energy prices dominate, partial insulation is an option.
- Longer term planning and regulation has a crucial role if small-scale bioenergy projects are to succeed.
- Flexibility and diversity can also reduce producer risk.
- Collaboration in the market chain is key at start-up.
- Long local market chains spread out the benefits.
- Moving bioenergy resources up the energy ladder adds value.
- Any new activity raising demand will raise prices, even those for wastes.
- Cases do not appear to show local staple food security to be affected.
- Small-scale bioenergy initiatives can offer new choices in rural communities.

Experience with bioenergy in Africa, including positive as well as cautionary examples, is presented in the section entitled Experience with bioenergy in Africa. As considered in more detail in the section entitled The Brazilian experience, Brazil provides a prominent example of simultaneous and apparently synergistic advancement of large-scale bioenergy production, food security, and economic well-being.

As a consequence of the continent’s very large land area, some of the most remote places on earth are in Africa. African agricultural producers far from ports and trade centers face the “double penalty” of lower prices for their products and higher costs for fuel and other inputs. In the 40 years preceding 2010, per capita world food production grew 17%, while in Africa it fell 10%, as population growth outstripped agricultural output [16]. One of the big problems faced by African farmers is the steep cost of transport, which means that African farmers pay two to six times the global cost of fertilizers [16]. Local production of bioenergy (heat, electricity, and biofuels for transport) to power farm machinery, dry and safely store crops, and enable transportation of goods to market could substantially alleviate this double penalty. It is notable in this context that diesel engines used in tractors and trucks can be powered by established biofuels, including not only biodiesel but also ethanol in the form of “E95” (personal communication, Jonas Stomborg, Scania).

Losses in the food supply chain, both in quantity and quality, exacerbate chronic food insecurity and malnutrition in Africa. The Food and Agriculture Organization (FAO) [17] estimates close to one-third of the world food supply to be lost in the supply chain. These losses occur at every step of the food supply chain, including harvesting, processing, preservation, storage, transportation, and cooking. Poor access to energy is among the most important factors responsible for these limitations. By improving such access, bioenergy development could play a crucial role in preventing crop and food losses.

A multitude of factors conspire to make it difficult for African farmers to sell crops competitively into world markets, as elaborated in compelling detail by Thurow and Kilman [18]. North America and Europe export large amounts of subsidized food at prices difficult for African farmers to compete with. However, these regions do not export biofuels and are unlikely to do so in the future, and exporting heat and electricity is not feasible. Thus, energy provides a potential catalyst for socio-economic advancement in Africa that is largely independent of several important factors that have made this difficult in the case of food production.

Government subsidies, international trade agreements, and other factors have led to relatively stable markets for producers and supply for consumers in developed countries. The consumer in the developed world, where distance between producer and the table has little impact, seldom notices regional droughts and transient decreases in production. In contrast, their counterparts in the developing world are much more vulnerable to even slight fluctuations in weather patterns or factors such as the availability of transport, fuel, and electricity. Typically, in years of abundance they do not have sufficient markets for their produce nor the means to store their produce, consequently leading to widespread spoilage and falling producer prices. But on multiple occasions oversupply has been followed by famine and skyrocketing prices in less than a year, with Ethiopia in 2003 and 2004 a notable example [18].

The precarious nature of food supply in Africa has often led to dependence on foreign aid. Yet the drivers for transformation on the African continent cannot be based on policies and regulations designed for the market-based Western economies. They also cannot be dictated by the food versus fuel debate that takes place in countries where food waste occurs not because of lack of transport infrastructure or storage facilities, but because of excess and consumer preferences, thus primarily at the retail and consumer level.

Any bioenergy strategy must be reconciled with the potential for collision between bioenergy feedstocks and food on a continent where an alarming fraction of the population is undernourished. Advancing bioenergy at the expense of food security is an unacceptably bad trade for Africa. There is increasing acceptance that bioenergy production and food security need not be in competition and could be complementary [11,19-24], but that is not the same as saying that food-fuel competition will not happen. Commenting on biofuels and local food security in developing countries, Locke and Henley [25] observe that

- Few studies use or attempt to measure the balance of all four pillars of availability, access, utilization, and stability of food.
- Available evidence does not provide a robust basis for a strong statement about the impact of biofuel projects on local food security in developing countries.
- The impact of biofuel feedstocks on food security may be similar to that of other commercial crops. It is not necessarily the fact that it is a biofuel feedstock that matters. What seems to matter is the production model used; the timing of impact measurement; the profitability of production; and the terms and conditions under which entitlements to land, wages, and prices are defined and productivity is raised.

Evaluating the effect of bioenergy on indicators of food security is somewhat different from evaluating the impacts of bioenergy on the causal factors that give rise to food insecurity, which include poverty, lack of economic development, and also physical, institutional, and market infrastructure [26]. Both evaluative frameworks are important, with the potential benefits of bioenergy likely more apparent in the latter.

Bioenergy is prominently featured in low-carbon global energy scenarios, for example, representing an average of 25% of primary energy supply in five scenarios compiled by Dale *et al.* [27]. Africa, today a small contributor to greenhouse gas emissions, has in many locations abundant resources to develop low-carbon bioenergy without having to compete with an established fossil energy infrastructure. Being the last continent to develop an economy based on fossil resources is unlikely to be a wise strategy for Africa. If unwisely deployed, bioenergy could make adaptive responses to climate change more difficult in Africa and elsewhere [28]. However, bioenergy can be an asset for such responses if wisely deployed. At a continental scale, substantive impacts from climate change are expected on Africa’s cropping systems, with severe high temperature episodes and increasing frequency and severity of droughts and floods potentially causing catastrophic failures in production [29]. Indeed, yields in many important staple crops, such as maize, rice, and wheat, in Africa are increasingly volatile and in a number of cases in decline [30]. At a local level, predicting the consequences of climate change remains highly uncertain [29]. Bioenergy systems should therefore be deployed in ways that support resilience (economic and climatic) in African food cropping by, for example, enabling economically productive novel crop rotations and cropping patterns to combat increasing levels of pests and diseases in both food crops and forestry systems [31,32] and alternative markets during times of oversupply [26].

UNEP has estimated that more than a quarter of the African continent is at present in the process of becoming useless for cultivation due to degradation [33]. Cultivation of perennial grasses, which are potential bioenergy feedstocks, is well established as a means to increase soil carbon stocks and restore degraded land [34-36]. However, this subject has in general received more study in temperate climates than under conditions typical in Africa.

In pursuit of maximizing the development benefits of bioenergy, it is important to consider the entire bioenergy supply chain. At the front end, the availability of land and means by which land is accessed are critical [25]. At the back end, the extent to which bioenergy products are - or are not - aligned with and used to address high-priority social needs is equally important. We note in this context that electricity, cooking fuel, and fuel for agricultural machinery are key needs in many parts of Africa, whereas the need for fuel for light-duty vehicles is often less critical. In situations where bioenergy can provide previously missing links that enable new value chains, there is potential for large and indeed transformative development benefits.

## Bioenergy overview

There are a substantial number of bioenergy feedstocks, conversion processes, and products, as summarized in Tables 1 and 2 and reviewed in more detail elsewhere [37,38]. Established combinations include:

- Woody cellulosic biomass undergoes combustion to produce electricity and heat.
- Starch- and sugar-rich crops undergo fermentation to produce ethanol.
- Oil seeds undergo pressing and transesterification to produce biodiesel.

**Table 1:** **Bioenergy feedstocks**

**Crop category**	**Example**	**Industry status**	**Land, environment, and energy**
Starch-rich^1^	Maize, wheat, sorghum	About 50 billion L ethanol in the US based on maize	Typically grown on high-quality cropland with substantial fertilizer input. Fossil energy displacement ratio 1.3 to 1.7. 4,000 L ethanol/ha in the U.S.
Sugar-rich^2^	Sugarcane, sugar beets	About 23 billion L ethanol in Brazil based on sugarcane	Grown primarily on former pastureland in Brazil. Agrichemical inputs less than maize. Fossil energy displacement ratio about 8 to 10. 6,700 L/ha in Brazil today, could be substantially higher with conversion of cellulosics, energy cane.
Oil-rich^3^	Rapeseed, soy, sunflower, palm oil	About 23 billion L produced worldwide, most in the EU, US, and Brazil	Rapeseed, soy generally grown on cropland. Most palm oil plantations are on former forests. Fossil energy displacement ratio 2 to 2.5 for rapeseed and soy, about 4 to 8 for palm. 530 L/ha for soy in Argentina; 3,600 L/ha for palm in Malaysia.
Cellulosic^4^	Grass, trees, various wastes	331 TWh electricity globally. Liquid fuel capacity about 175 million L worldwide	Could in principle grow on land unsuitable for crops. Potential environmental benefits when incorporated into agricultural landscapes. Fossil energy displacement ratio somewhat speculative for liquid fuel production but expected to be similar to sugarcane. Over 7500 L/ha based on miscanthus yields in the US (25 tonnes/ha), 75 US gal/ton.

^1^Starch-rich crops: annual production [39]; fossil energy displacement [40]; corn yield [41]; dry mill yield [42].

^2^Sugar-rich crops: annual production [43]; fossil energy displacement and ethanol land yield [44].

^3^Oil-rich crops: annual production [7]; fossil energy displacement [45,46]; soy oil yield [47]; palm oil yield [48].

^4^Cellulosic crops: global electricity [49]; global cellulosic biofuel capacity [37]; current miscanthus yields [50].

**Table 2:** **Modern bioenergy conversion technology summary**

**Category**	**Products**	**Technology**
**Non-biological**		
Combustion^1^	Electricity, heat	Mature. Electricity generation rather capital intensive (about $1,900 - $4,300/installed kW).
Gasification^2^	Electricity (via gas turbines) or synthetic gasoline and diesel (e.g., Fischer-Tropsch)	Limited commercial application. Often highly capital intensive (about $375/L annual capacity for coal liquefaction in South Africa).
Pyrolysis^3^	“Biocrude”, a mixture of liquid-phase organics	Limited commercial application. $2/L annual capacity for production of naptha and diesel.
Pressing and transesterification^4^	Biodiesel from oil-rich crops	Mature. Relatively simple, low capital ($0.33/L installed capacity for biodiesel in Europe).
**Biological**		
Fermentation of starch and sugars	Ethanol, potentially many other molecules	Mature for ethanol production. Capital cost^5^ about $1.20/L installed capacity for ethanol with cogeneration in Brazil, about $2/L installed capacity for maize ethanol in the US.
Anaerobic digestion	Methane	Rather mature. Can be applied to both liquid and solid wastes. Many thousand small-scale digesters operative, particularly in China and Germany.
Lignocellulose hydrolysis and fermentation	Ethanol, potentially many other molecules	Not mature. Hydrolysis can be accomplished via acid or enzymes. Several fermentation options and configurations.

^1^Combustion capital costs: [51].

^2^Gasification capital costs: [52].

^3^Pyrolysis capital costs: [53].

^4^Biodiesel capital costs: [54].

^5^Sugarcane ethanol capital costs: [55].

Processes based on grains, sugarcane, or palm oil achieve rather high per hectare fuel productivity. However, this parameter is generally lower for fuels from oil seeds, which are in many cases coproducts of animal feed production. Fossil fuel displacement ratios, as well as greenhouse gas emission reductions, are generally high for processes based on sugarcane, cellulosic feedstocks, and oil-rich crops, and positive but moderate for bioenergy production from grains. Processes based on cellulosic feedstocks offer broad site range, potential for high per hectare yields, and low feedstock purchase cost. In addition, there is well-documented potential for environmental benefits from incorporating perennial grasses into agricultural landscapes with respect to soil fertility and land reclamation, water quality, and wildlife habitat [34,35,56-58]. While cellulosic feedstocks are widely thought to offer great promise for the future, conversion technology to liquid fuels is still under development and is not yet widely applied.

The potential of drought-resistant plants in regions with lower precipitation should also be considered. For example, agave plants are drawing attention as a prospective feedstock for biofuel production because of their ability to grow in dry climates, high biomass yield, and high concentrations of soluble sugar content [59]. A recent life cycle analysis of the potential of these succulent plants as a feedstock for first-generation biofuel production suggests that they show much promise with minimal impact on food production or pressure on water resources [60]. Traditionally, agaves are commercially cultivated primarily as a fiber source, often in arid, warm regions; some can tolerate temperatures of up to 65°C [61] and are therefore a good feedstock candidate for second-generation biofuels in an African context, where residues could potentially be further processed in small-scale operations for heat or electricity generation. Another intriguing aspect of some of the agaves is their response to increases in CO_2_ concentration. Graham and Nobel [62] performed long-term experiments that showed a greater than 100% increase in water-use efficiency and a significant increase in dry mass production when the CO_2_ concentration was doubled.

Compounding new technology risks with risks likely inherent in many African applications - for example, those involving infrastructure, business models, and governance - is unlikely to be a good strategy. As a result, a strong argument can be made for deploying established bioenergy technology in an African context. At the same time, improvements in technology for both biomass production and conversion may make possible more beneficial and widespread application in the future. Considering these two factors together, it is important to employ meritorious, current bioenergy technologies in ways that enable rather than impede deployment of future technologies, and to develop and deploy future processes in ways that expand rather than contract opportunities for early adopters and investors [63].

An illustrative and potentially important example is the possible progression from established processing of sugarcane to not-yet-established cellulosic biofuel technology. Sugarcane processing to ethanol, often accompanied by electricity and/or sugar, produces fuel competitive with global petroleum prices, has a very positive ratio of fossil fuel displacement: fossil fuel input, high fuel yields per hectare, and generally positive sustainability metrics [64,65]. Lignocellulose is present in sugarcane in about a 2:1 ratio relative to sugar. Converting the lignocellulose as well as the sucrose fractions in sugarcane would substantially increase yields of energy and revenue per ton, and growing “energy cane” with reduced sugar content would have the multiplicative effect of increasing tons per hectare. Once conversion of the lignocellulose component of sugarcane is established, this would enable conversion of other cellulosic crops, for example, those with a higher tolerance to drought, that could be grown where sugarcane cannot. Thus, there is a continuous and potentially advantageous path from fermenting only the soluble sugars present in cane to also fermenting cellulosic residues once the required conversion technology is available.

Bioenergy can and is being produced over a broad range of scales from village-scale digesters and biodiesel refining operations to industrial-scale facilities which produce up to a half billion liters per year of fuel and process up to over five thousand dry metric tons per day of feedstock. Large-scale facilities require large land areas as well as technological expertise and capital not available within many African communities. At the same time, high efficiency and financial viability are often easier to achieve at a larger scale as compared to smaller scale and scattered markets with low purchasing power of the populations. This conundrum remains to be resolved, and is likely to be fertile ground for creative approaches that are tailored to location-specific circumstances and will likely evolve over time. The Brazilian experience suggests (see the section later in the paper) that broadly distributed social benefits and large-scale efficient bioenergy production need not be mutually exclusive.

## Experience with bioenergy in Africa

**Figure 2:**
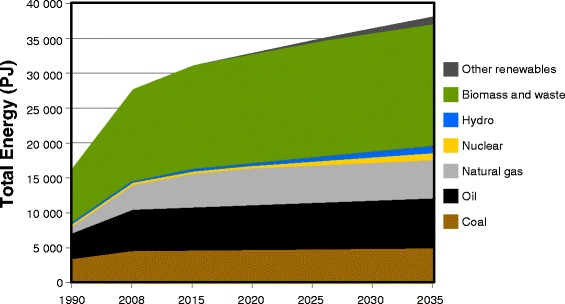
**Total primary energy demand for energy sources on the African continent, 1990 to 2035 [** 66 **].**

In 1990, Africa’s primary energy consumption had reached 16 EJ, less than 5% of the global energy demand, of which bioenergy provided 60%. By 2010, its primary energy consumption had risen to 28 EJ, slightly more than 5% of the global demand, with bioenergy providing about half of this for the continent as a whole and much larger shares in some regions [66]. Africa’s dependence on traditional forms of biomass for energy has not diminished and is not predicted to do so in the foreseeable future (Figure 2).

Biomass has been and remains the main source of energy for many people in Africa both in rural and in urban areas. For Sub-Saharan Africa (excluding South Africa), over 80% of the total energy supply for heating, cooking, and processing of agricultural produce is derived from biomass, such as fuel wood and agricultural residues [66,67]. In most of this region’s cities, where the population is still booming, the majority of households are dependent on wood energy more than on any other sources for such purposes. Cooking on open fires is highly energy inefficient and also poses a major public health problem; an estimated four thousand Africans die prematurely every day from household smoke pollution [67,68]. Demand for wood for cooking, particularly when converted to charcoal to sell into urban markets, can exceed supply, resulting in environmental degradation in addition to serious health impacts [67,68]. By contrast, modern bioenergy involves using higher efficiency technology to produce fuels, electricity, and heat.

Africa is looking for more efficient and affordable household energy sources that can enhance rural development and reduce the burden on women to provide the energy needs of their households while combating deforestation, land degradation, and desertification. In this context, there have been various bioenergy initiatives implemented to increase access of rural and peri-urban populations to clean and sustainable energy and modern bioenergy sources. These initiatives have targeted both the demand and supply sides. Projects can be categorized as follows:

1. Increasing access to traditional sources of energy such as wood and charcoal in a more sustainable manner through reforestation and investments in energy production plantations while increasing diversification of products and income opportunities on the end-user side, and the use of efficient conversion technologies such as improved cookstoves. Examples include projects funded by the World Bank in the Democratic Republic of Congo, and in Malawi with the Jatropha, Neem, and Moringa project [67,69,70].
2. Using agricultural residues, municipal waste, and non-food crops, hence avoiding competition with food crops. Such energy sources are not fully developed and constitute a promising avenue, as demonstrated through several experiences in various regions of the continent. Country-specific projects include those in Senegal, Ghana, Kenya, Uganda, Tanzania, and Malawi, as presented in Table 3.

**Table 3:** **Examples of bioenergy initivatives in Africa**

**Country**	**Initiative**	**Opportunities/comments**
Ethiopia	National biogas program, which plans to build 14,000 domestic biogas digesters [71]. A 5% blending of petrol and ethanol since 2008.	Under the national biomass program, a 4-year demonstration project has demonstrated notable benefits of replacing fuelwood (currently 29%) and kerosene (42%) with ethanol stoves; notably reduced foreign exchange to import kerosene, reduced distance traveled to collect firewood by 73%, and improved indoor air quality [15].
Ghana	Jatropha oil for mixing with diesel (70% plant oil/30% diesel) to fuel butter processing equipment, and as a kerosene substitute for use in lanterns [72].	Village-level biofuel production. *Note:* Jatropha has been planted in a number of other African countries such as Malawi and Mozambique (see below) as well as Mali [15]. In South Africa currently only allowed for experimentation [73].
Kenya, Tanzania, and Uganda	Afforestation for sustainable charcoal production [74].	Charcoal making supports about 500,000 full-time and part-time charcoal producers. Wood fuel demand is double the supply, with forest cover decrease by 2% annually, thus incentive for tree planting. Charcoal remains preferred choice over briquettes despite higher price and more pollution. *Note:* See also initiatives in Senegal [15].
Madagascar	Ethanol as a household fuel and alternative sources of energy to relieve the pressure on forest resources and reduce childhood mortality [75].	Identified need for a regulation, Government support and optimization identified as key requirements for success.
	Gel fuel to replace charcoal as a cooking fuel in urban areas [69].	Identified need for economic sustainability.
Malawi	Restoration and commercial use of tree crops, including marginal lands [70].	Potential for integrating various tree species to increase crop yield, rehabilitate degraded land, and improve the soil fertility. Products are used as bio fertilizer and green charcoal.
Mauritius	Cogeneration, primarily using bagasse, renders sugar industry electricity self-sufficient, with estimates that excess bagasse-derived power accounts for 30% of total electricity demand in the country [76].	Life cycle analysis shows that despite potential negative consequences such as high water consumption and eutrophication, benefits include lower GHG emissions and acidification; probably the only stable alternative to 100% coal imports.
Mozambique	Initiated in 2004, biofuel production originally dominated by small-scale farmers, now by foreign commercial investors [77].	Originally the focus was primarily on jatropha biodiesel, now there is increased emphasis on bioethanol derived from sugarcane and sorghum.
South Africa	Mandatory blending of petrol and diesel with biofuels as follows: 5% minimum concentration for biodiesel blending, and permitted range for bioethanol blending from 2% to 10% v/v [78]. Target date of 1 October 2015.	South African Airways plans 50% use of aviation biofuels by 2020. Energy crops include sweet sorghum and sugarcane [79,80]. Renewable energy feed-in tariff implemented to establish energy prices including a profit margin to attract developers to invest [81].
Tanzania	Sisal biogas. Conventionally only 4% of the plant (fiber) has been used to make items such as ropes and carpets. Two projects to date resulted in improved efficiency for biogas and biofertilizer production; current electricity output is150 kW with plans to expand to other estates for a total of 6 MW [15].	A private company without external support leads this initiative, which led to an 80% increase in the number of children attending school, while access to health care also improved as a result of the energy supplied to schools and hospitals.
Zimbabwe	Planned current 5% blending of ethanol in petrol to 15% [82].	The technical feasibility and potential were demonstrated when the commercial producer reached maximum generation capacity of 18 MWe. About 8 MWe is used for sugarcane ethanol, leaving 10 MWe surplus.
	Jatropha cultivation for biodiesel [83].	Objective is to produce biodiesel to meet 10% import substitution (approximately 100 million L per year) from jatropha, using an existing facility operating on cotton and sunflower seeds.
3. Using liquid biofuels such as ethanol and biodiesel and the corresponding technologies for conversion and utilization to substitute the traditional sources and conversion technologies. This is the case in the Ethiopian government-led project, but also in several other Southern and East Africa countries including Madagascar, Mauritius, South Africa, Zambia, and Malawi, to name a few. Examples of these options and related initiatives are summarized in Table 3.

Diaz-Chavez [20] reported a detailed study of biofuel development and potential in African countries selected to represent different regions: Senegal, Mali, Kenya, Tanzania, Mozambique, and Zambia. This study concluded that Africa has potential to meet both its food and fuel needs from biomass, neither of which occurs today, and that biofuel production could help unlock Southern Africa’s latent potential and positively increase food production if it brings investment in land, infrastructure, and human resources. Further conclusions, illustrative of both potential and challenges, included the following:

- Yields of currently cultivated land in the less developed countries could be tripled by using improved management practices, potentially freeing up more land for biofuel production.
- It is estimated that the area under sugarcane in the region could be doubled without reducing food or destroying valuable habitats.
- Mozambique has immense agricultural potential, with an estimated 36 million ha of arable land of which only 10% is presently in productive use.
- Negative impacts have occurred in some areas (not whole countries), such as displacement, and these should not only be avoided but legally penalized.
- The capacity to implement and monitor needed policies is limited in some countries.
- Bioenergy projects in Africa have not been without challenges related to feedstock production, technology, and social factors such as consumer preferences and institutional coordination. In particular:
- There is a constraint of reliable feedstock supply under circumstances that achieve low agricultural yields today. Given the low and/or volatile level of yield for many crops - most of which are rain fed with low access to quality inputs and equipment - bioenergy projects have suffered from irregular feedstock provision in terms of quality and quantity, making the availability of bioenergy products unstable and unpredictable. When feedstocks are derived from non-food crops for which a research gap remains to be filled, for example, jatropha or other tree crops, the situation has often been particularly challenging. Under such circumstances, price stability and confidence of consumers are easily eroded, and the new adopters shift back very quickly to traditional biomass sources of energy and equipment, for which sources of supply are well established. The myth that some favored new crops, such as jatropha, would be immediately commercially productive on marginal land is now realized to be predominantly false [9].
- Consumer preferences are difficult to shift to new technologies in cases where the energy density and efficiency of new biomass-derived products is lower than that of well-established products. On the other hand, ease of handling, including safety and cleanliness, have been found to be a significant factor for adopting liquid-based biofuels such as ethanol for cooking [74].
- Experience in many African countries reveals that price incentives have not been sufficient for adoption of biofuels given the lower energy density of the new product (briquettes for instance) compared to charcoal. Under such circumstances, more research is needed in order to improve the efficiency of these new technologies.
- Isolated projects, even those with tangible outcomes, have in some cases not proved sustainable or conducive to a qualitative transformation process. This has been the case in a number of projects conducted by external partners with weak involvement of government and national stakeholders. Furthermore, many projects still need to be scaled up for a real impact on a large fraction of the population.
- Institutional constraints must also be faced in terms of coordination and synergies to be built among government units. Agriculture, environment, and energy departments rarely work together to discuss and design bioenergy strategy frameworks and harmonized policies and regulations. Private sector participation is also at its early stage, as most projects are initiated by non-governmental organizations (NGOs) and international partners.

Although modern bioenergy industries are emerging in several African countries, in particular, where an incentive exists for blending ethanol with gasoline, most of them still lack the capacity to develop an economically viable and sustainable bioenergy industry. However, opportunities exist as several regional economic communities have defined very clear strategies that need substantial support to be adapted and implemented in a comprehensive manner at the national level. This is the case, for example, of the West African Economic and Monetary Union, which has adopted a bioenergy strategy since 2008 [84]. One of the main drivers of bioenergy development in this region resides in reversing the trend of desertification and land degradation, and developing sustainable energy sources for cooking, heating, and food processing. Therefore, the key strategies aimed at providing alternative fuels can be expected to benefit from reliance on a combination of feedstocks provided through reforestation with fast-growing and adapted species that can be harvested sustainably and processed into cleaner fuels. In areas where reforestation is not possible, bioenergy development has been encouraged through multicropping systems and careful management of water resources [84].

## The Brazilian experience

Brazil’s modern bioenergy industry, one of the two largest in the world in absolute terms, is by far the largest in terms of fractional energy supply, and is the foremost example of bioenergy deployed in a developing country context. Soils and climates in much of Africa have similarities to those in Brazil, and Africa and South America are widely recognized as the continents with the greatest potential to increase modern bioenergy production [85]. Over the last three decades, Brazil saw marked increases in social development (minimum wage increase, poverty and hunger reduction), went from being a small player in international agriculture to the largest exporter in the world (number one in soybeans, beef, chicken, oranges, and coffee), and became energy independent with a large contribution from modern bioenergy (Table 4). There is substantial evidence that the emergence of Brazil’s bioenergy industry positively impacted simultaneous advances in social development and agriculture. Brazil’s bioenergy experience is thus of distinctive relevance to Africa.

**Table 4:** **Summary of Brazil’s advances in social, agricultural, and energy sectors: 1980 to 2010**

**Sector**	**Social**			**Agricultural**	**Energy**		
**Index**	**Minimum wage (US$**_**2010**_ **/month)**	**Population out of poverty (%)**	**Global hunger Index (GHI)** ^**a**^	**Exports (million US$**_**2010**_ **)**	**Net imported (% supply)**	**Renewable (% supply)** ^**b**^	**Biofuels (% liquid fuel supply)**
	[86]	[87]	[88,89]	[90]	[91]		
1980	207	67	10.4 (1981)	24,700	42%	46%	8%
2010	298	90	4.0 (2011)	62,100	10%	47%	27%

^a^The Global Hunger Index is used to evaluate the hunger situation by countries, considering: a) the undernourished population as a percentage of the total population, b) the prevalence of underweight children under the age of 5, and c) the under-5 mortality rate. Values less than 4.9 reflect “low hunger”, values between 5 and 9.9 reflect “moderate hunger”, and values between 10 and 19.9 indicate “serious hunger”. The worst global hunger scores in 2011 were ascribed to Burundi and the Democratic Republic of Congo, with scores of 37.9 and 39, respectively.

^b^The share of renewable energy supply remained about constant, but shifted from wood fuel used in households for cooking to liquid biofuels used in the transportation sector. In this period the total energy supply increased 234% (115 to 269 Mtoe) [91].

However, we acknowledge at the outset the tremendous diversity of circumstances on the African continent, and that the Brazilian bioenergy model will in most cases require some adaptation to these circumstances. We note that development of bioenergy in Brazil has until recently targeted national markets, which for some African countries are small and/or otherwise impractical to rely on. As well, the expansion of Brazilian bioenergy production seen since 1980 began with already established industrial production of both sugar and ethanol, thereby providing a foundation of expertise and purchasing power that are present in some but by no means all African countries.

Sugarcane has been cultivated in Brazil since the sixteenth century and has always represented an important economic activity. In 1931, aiming to reduce dependence on imported liquid fuels and absorb the excess production of the sugar industry, the Brazilian government implemented a compulsory blend of at least 5% anhydrous ethanol in gasoline. During the period from 1931 to 1975, an average of 7.5% of gasoline demand was met by ethanol. In order to further reduce oil imports and increase energy security, the Brazilian government created the National Alcohol Program (Proálcool) in 1975. This program has evolved since then, with ethanol reaching price parity with gasoline on a BTU basis in about 2005 [65]. A particularly significant development was the introduction of flex-fuel cars, able to use any blend of gasoline (E25) and hydrous ethanol. Flex-fuel cars currently represent 95% of sales of new cars, and pure ethanol can be used by 12.7 million Brazilian vehicles representing 47% of the national fleet [92]. Ethanol currently provides about 50% of light-duty fuel and 25% of total road transport fuel in Brazil, with biodiesel production about one-tenth that of ethanol [91]. However, the growth of ethanol production in Brazil has stalled in recent years due to government policies that maintain lower-than-market gasoline prices [93]. Ethanol production as practiced today in Brazil has generally positive sustainability indicators, notably including life cycle greenhouse gas emissions on the order of 10% of a gasoline base case [94].

As in many other countries, Brazilian mills processing sugarcane use bagasse to produceheat and electricity. Increasingly, surplus electricity is sold to the grid. Today bagasse is the second leading source of energy for electricity generation in Brazil after hydropower [91]. The progressive introduction of more efficient cogeneration systems allowed surplus electricity per ton of sugarcane processed to increase from approximately 20 kWh to up to 140 kWh in the most efficient mills, with room for further improvement to reach to about 200 kWh via integrated biomass gasification and combined cycles [95]. The electricity produced in Brazil from bagasse in 2012, 25 TWh, represents 5.6% of the electricity consumption in Brazil [96]. The installed power generating capacity of cogeneration systems in Brazilian mills, 9.3 GW, is a third of the 28 GW of installed capacity in the 47 Sub-Saharan African countries excluding South Africa [97]. Development of electrical generating capacity from bagasse in Brazil is a relatively recent event, occurring entirely within the last decade. As previously mentioned in Table 3, cogeneration from bagasse in Mauritius is extensive.

**Figure 3:**
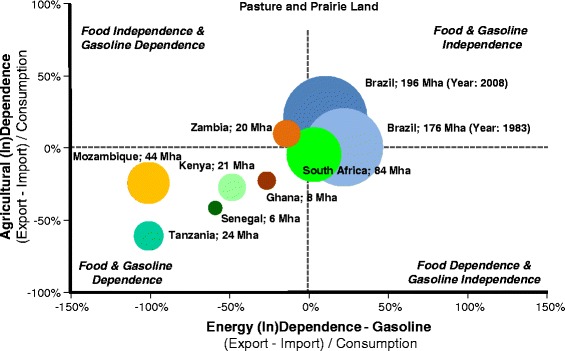
**Agriculture (in)dependence and gasoline (in)dependence and pastures/prairies area [** 89 **,** 91 **].**

It is interesting to stress the relevance of yields improvement and densification to reduce the land requirement for agriculture, including bioenergy production, in Brazil [98]. In recent decades, the sugarcane yield (tons/hectare) grew at a cumulative average annual rate of 1.4% and the process yield (liters ethanol/ton) grew at an average rate of 1.6%, resulting in an average annual increase of 3.1% in ethanol production per hectare. Thanks to these gains, the area currently dedicated to the cultivation of sugarcane for ethanol production is 38% of the area that would have been required to obtain such production with the yields observed when Proálcool began. Almost all of the 4.8 Mha used to produce ethanol in Brazil, representing about 1.3% of the total area of rural properties, is former pasture land. Over the lifetime of the Proálcool program, pasture land devoted to beef production has decreased by 10%, but beef production has more than tripled as a result of both higher stocking densities (head/ha) as well as higher animal performance (kg beef/head/year). Roughly threefold yield gains have also been observed over this period for grains and maize [99]. As shown in Figure 3, Brazil has achieved both food and gasoline independence, whereas substantial reliance on imports is observed for several African countries with substantial land resources.

There are about 400,000 direct jobs specifically related to ethanol production in Brazil, excluding the workers associated with sugar production [100]. Under current conditions, the production of bioethanol per unit of energy produced, compared with mineral carbon, hydroelectricity, and oil, requires, respectively, 38, 50, and 152 times more human labor [44]. About 81.4% of the employees work under a formal labor contract, compared to about 40% in the Brazilian agricultural sector as a whole. Formal work relations assure legislatively mandated rights such as retirement and annual paid vacations, unemployment insurance, extra monthly wages per year, health programs, and improved work conditions. Cooperative relations with workers’ unions where sugarcane mills operate has fostered, among other benefits, reduction of illiteracy and increase of years attending school, and a reduction in underage workers (from 15.3% in 1981 to less than 0.3% in 2009 [100].

In a detailed analysis of the socio-economic impacts caused by the expansion of sugarcane cultivation, Assato and Moraes [101] studied the results of establishing sugarcane processing plants in two municipalities, Nova Alvorada do Sul and Rio Brilhante. They found an increase in the aggregate income which boosted local markets, as evidenced by an increase in the number of shops and services as well as a more active real estate sector. They also noted that jobs which derived from the expansion of the sugarcane industry, and from other industries related to this activity, have played a key role in retaining and attracting residents, thus reducing rural exodus and contributing to increased population in the two towns they analyzed. These towns feature a large number of surrounding rural settlements, in which crops are cultivated that existed before the arrival of the sugarcane industry. Assato and Moraes observed that the income (often subsistence initially) of family farms in these settlements was supplemented with the wages from the jobs created by the sugarcane industry either in the ethanol plants or in the sugarcane fields. A significant portion of family farmers reported improvement in their quality of life due to the social programs offered by the sugarcane industry-related companies and due to opportunities for (re)training, employment and education, especially for children. Data collected from interviews indicated improved education during the period after the installation of the sugarcane industry. The authors conclude that introduction of sugarcane culture created jobs that led to an increase in aggregate income of the municipalities, and through multiplier effects enabled improved indicators of health, education, and quality of life.

The question of how the Brazilian agricultural sector would have developed without the simultaneous rapid growth of the bioenergy industry is complex and would likely benefit from more study. Although bioenergy development was not a primary cause of the growth of Brazil’s agricultural sector, it has likely been an accelerating factor in light of contributions to the development of rural communities and human resources together with improvements in logistics and trading infrastructure. Social development, agricultural development and food security, and bioenergy development in Brazil have been synergistic rather than antagonistic.

Important lessons from the Brazilian bioenergy experience of potential relevance in the African context include the following:

1. It is valuable for bioenergy feedstocks to be well known in agricultural terms, taking into consideration regional factors. Support by breeding programs, built on a foundation of good germ plasm, is essential.
2. Selling into multiple product markets (for example, food, fuel, electricity) has been advantageous in Brazil.
3. Bioenergy production chains should score well in terms of life cycle indicators, which are generally fostered by efficient use of land, water, and energy.
4. The state and its agencies have fundamental roles in fostering sound biofuel programs by assessing/creating/monitoring/enforcing the conditions for production/use, preferentially within a clear legal and normative framework. Important tasks include defining fuel (and blends) specifications, setting mandatory blending levels and implementing the program, and establishing a balanced tax regime taking into account appropriate externalities. These tasks are complex and demand both a technical background and negotiation among stakeholders, who frequently present contradictory perceptions and aims.
5. Social benefits should be explicitly considered within an integrated framework that also considers commercial viability, and are generally fostered by efficient production chains (point 3).

## The evolution of African agriculture

Van Kuelen and Schiere [102] suggest a scheme for the evolution of agriculture, focusing on mixed farming systems. Borrowing heavily from the four-stage progression they outline, we adapt this scheme here to describe agriculture in general and present attributes of each developmental stage.

**Figure 4:**
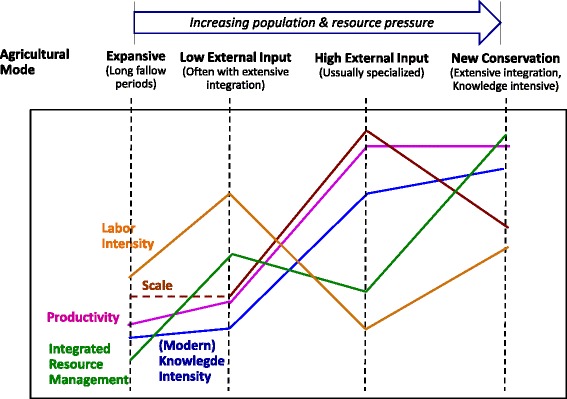
**Evolution of agriculture.**

As depicted in Figure 4, increasing population and resource pressure drives agriculture through a progression of modes from expansive/long fallow, to low external input/highly integrated, to high external input/specialized, to new conservation agriculture featuring extensive integration and high knowledge intensity. Agricultural integration, involving the exchange of material and energy between various agricultural activities and in particular crop and livestock production, plays a central role in this progression.

Most of Africa is supported by low input agriculture. Integration is practiced widely in some locations, for example, raising animals and crops on the same land in different parts of the year. However, the scope for integration can be restricted somewhat by very small farm sizes, for example, one or two hectares. Although much of the world’s effort to increase food productivity is focused on specialized agriculture with high inputs, 50% of the world’s food production and 70% of the world’s people are supported by mixed crop-livestock agricultural systems featuring a significant level of integration, and much of this agriculture involves low inputs [103]. Just as cell phones proliferated within Africa, bypassing the need to build a network of wires and poles, we see potential - and many benefits - to Africa progressing from the low external input, often integrated, mode to an African brand of new conservation agriculture bypassing some aspects of the high input/specialized mode. Realizing this potential is a challenge for policy makers, as we partially address in the section entitled Future directions.

Much has been written about bioenergy production from food crops grown outside of Africa leading to higher food prices and compromised food security [104-106]. Considerably less attention has been given to impacts of modern bioenergy production in Africa and, in particular, the potential benefits of such production with respect to food security. In fact, very few African examples of modern bioenergy production have been in existence at either small or large commercial scales over a long enough duration for sufficient data to be available to draw robust conclusions. In the Scurlock *et al.* [107] analysis of the relatively large-scale Triangle sugarcane ethanol plant in Zimbabwe, mainly benign and positive impacts on sugarcane production and productivity were found by the implementation of an ethanol plant annexed to a sugar mill.

Perhaps more speculatively (and we acknowledge, controversially), it is possible to foresee an important role for biofuels in supporting resilience in food cropping, as opposed to the competitive outcome with food provision and access that is most often assumed. Here we speculate what might have happened over the last decade if Zambia, and indeed South Africa, had implemented a large-scale biofuel production program based on the use of maize as the primary feedstock. Crop production in Sub-Saharan Africa can be described as a boom, but under-supply, cycle that can lead to bouts of severe undernourishment. For example, in Zambia during 2010 and 2011, as a result of adverse climate conditions the maize crop failed, and Jayne [108] states that ”the government of Zambia spent 2-3% of GDP stabilising food prices. In 2012, better climatic conditions returned and a 1.5 million tonne maize surplus was generated. However, as the country only had the capacity to export 70 000 tonnes per month to other countries, it would have taken ‘20 months to export the surplus by which time (as a result of a lack of storage infrastructure) most would be unsuitable for human consumption. “Similar cycles are seen throughout the continent. And yet, if Zambia had a biofuel industry capable of using all or part of the grain surplus, an economic take-off would have been available supporting the development of the production and storage infrastructure, and during times of crop failure the remaining crop could be diverted back to human food markets. In this way the maize supply chain could become more resilient to climate shocks.

## Future directions

Modern bioenergy can be an agent of African transformation, with potential social benefits accruing to multiple sectors and extending well beyond energy supply per se. Potential negative impacts also cut across sectors. Thus, institutionally inclusive multi-sector legislative structures will be more effective at maximizing the social benefits of bioenergy compared to institutionally exclusive, single-sector structures. This critical point is articulated well by the 2011 Practical Action working paper [9]:

“the role of government is to provide stimulus for private investment and initiatives, as well as promote effective regulation, monitoring and co- ordination of the biofuels sector. The particular multifaceted opportunity that liquid biofuels offers for Africa demands a new type of public, private and governmental engagement and integration, which may be very beneficial for Africa’s overall growth and development. Given the complexity of the different policy objectives, and the many unknowns, the industry is still more likely to succeed within a purpose built legislative structure, than within the current inadequate and/or conflicting frameworks. Subsequently, working with all relevant ministries and aligning policy within a clear dedicated biofuels policy is the best way to achieve sustainable results”.

Conceptual models for modern bioenergy deployment in Africa may be thought of along an axis defined by the extent of social engagement. At one end of this axis, which we term the “low social engagement model”, bioenergy feedstock production can be imagined in areas that are unoccupied and unused or nearly so, hence destined to consumers located outside of the area, that is, urban, regional, or export markets. At the other end, termed here the “high social engagement model”, feedstock production can be imagined in areas that are occupied and used to a considerable extent. In this case the business model can be either a cash feedstock crop or a local feedstock for local bioenergy development.

Pursuing deployment according to the low social engagement model is certainly simpler, may be beneficial in some instances, and could be a step in a sequence of actions leading to realization of development objectives. However, the potential development benefits of the high social engagement model are likely to be substantially greater. We note that occupied areas capable of growing energy crops are far more plentiful in Africa compared to unoccupied areas, which are in most cases degraded, dry, and/or landlocked and remote. Although difficult to quantify objectively, we offer the impression that considerably more effort has gone into analysis along the lines of “How much bioenergy could be produced once food needs were provided for?” as compared to “How much more food security and other social benefits could be realized with bioenergy than without it?” In the context of African development, we find the latter question to be considerably more compelling.

Although there is widespread awareness of a stunning gap between the actual and potential output of Africa’s land resources [14], and some important initial exploration has occurred (see the section Experience with bioenergy in Africa), there is much more to be done in the area of analyzing integrated scenarios featuring increased production of food and bioenergy. Table 5 presents a framework wherein the “What is?” and “What could be?” questions are examined from the point of view of geography, land management, society, environment, and synthesis, culminating in a vision for multiply beneficial land use.

**Table 5:** **Framework for development of a vision for multiply beneficial land use**

**Domain**	**What is?**	**What could be?**
a. Society	Wealth generation/distribution, and access to capital; supply and demand of food, water, fodder, and energy; land ownership and occupation	Define needs and aspirations based on community and stakeholder input at relevant scales.
b. Geography	Precipitation, temperature, soil texture, irrigation potential.	Define potential yields of food crops, pasture, and energy crops.
c. Land management	Land cover, use, and disturbances; current crop yields	Define how management would have to optimize the potential defined in domain b based on the needs and aspirations defined in domain a.
d. Environment	Inventory C and N? Flows, ecosystem services, soil and air quality, water quality and access.	Evaluate the changes in domain c with respect to environmental objectives; propose strategies to mitigate any conflicts.
e. Synthesis		Considering all aspects, develop a vision for multiply beneficial land use responsive to social and economic priorities featuring production of food and bioenergy without compromising water and other natural resources, and catalyzed by responsible investment.

The spatial scale chosen for analysis will impact the execution and outcome of efforts to develop a vision for multiply beneficial land use featuring production of both food and energy from the land. Analysis at the national or multi-national level will be informative with respect to operative federal policies and regulation, aggregated impacts, and consideration of integrated strategies and benefits at a high level. Analysis at the level of the feedstock catchment area for a single potential production facility will be particularly informative with respect to local circumstances, objectives, and benefits, and will be more relevant to potential projects. For many purposes analysis at both levels will be needed. Visions developed in different locations will likely have some features in common, but will also reflect the tremendous diversity of circumstances across the African continent.

Once a vision for multiply beneficial land use is developed, regardless of scale, the next step is to ask “What needs to be done to close the gap between *what is* and *what could be*?” The answers will in general be location-specific, and will usually involve a variety of players including communities, companies, federal and local governments, and NGOs. In many cases it will be useful to target the simultaneous realization of two goals: 1) sustainable and widely distributed social benefits and 2) commercial viability. Given this duality, there is significant scope for creative partnerships between the public, private, and NGO sectors. The impetus for such partnerships can be expected to result from further analysis of multiply beneficial land use.

Pursuing social benefits and commercial viability within the context of the high social engagement model for African bioenergy development might proceed via the following steps:

1. Develop a multiply beneficial land use vision and strategy along with appropriate and inclusive land tenure systems (see above).
2. Provide - either by government, companies, or public-private partnerships - adequate incentives in terms of facilitating access to input and output markets and mitigating investment risk for smallholder farmers to increase food and non-food crop yields. Note that several-fold increases have been observed to result from simple extension measures [109,110].
3. Investment would be gathered - by a company, co-op, or public-private partnership - to build a bioenergy conversion facility with bioenergy feedstocks planted on land made available by and fostered by an enabling environment and adequate incentives.
4. Monitor and optimize social benefits and environmental impact.
5. Share best practices within and across regions.

As elaborated conceptually in the section Bioenergy as a potential enabler of development, and supported by experience in both Africa and Brazil, as we have discussed, we see strong evidence that the benefits of proceeding to step 4 can be substantially greater than those achieved by stopping at step 2. That is, we think it is very likely that measures to advance food security and bioenergy development can be a substantially more effective development strategy when pursued together than either can alone.

In many examples of bioenergy deployment in developing countries, social consequences have been an afterthought rather than an integral part of project planning. Even when pursued in this mode, it appears that impacts of bioenergy on food security and economic development have in some cases been demonstrably positive, with the experience in Brazil being a prominent example. Still, some projects are more beneficial than others, there are examples of projects that have had negative impacts, and even projects with positive impacts for a majority are likely to have negative impacts on a minority that would be desirable to mitigate [25]. To the extent that development objectives become integral to project planning, the magnitude, probability, and distribution of anticipated social benefits from bioenergy rise markedly. Developing and implementing policies and institutional structures that foster such integration is challenging and very much a work in progress. Notwithstanding, the potential of bioenergy to positively impact Africa’s pressing challenges requires that it be urgently considered and advanced.

## Conclusions

Africa has the highest incidence of food insecurity and poverty and the highest rates of population growth, but it also has the most arable land, the lowest crop yields, and by far the most plentiful land resources relative to energy demand. In Brazil, social development, agricultural development and food security, and the development of modern bioenergy have been synergistic rather than antagonistic. Achieving such synergies in African countries will require clear vision, good governance, and adaptation of technologies, knowledge, and business models to myriad local circumstances. Strategies for integrated production of food crops, livestock, and bioenergy are potentially attractive and offer an alternative to an agricultural model featuring specialized land use. Modern bioenergy can be an agent of African transformation, with potential social benefits accruing to multiple sectors and extending well beyond energy supply per se. Potential negative impacts also cut across sectors. Thus, institutionally inclusive multi-sector legislative structures will be more effective at maximizing the social benefits of bioenergy compared to institutionally exclusive, single-sector structures. Innovative business models (such as public-private partnerships) aimed at maximizing social benefits are also promising. If done thoughtfully, there is considerable evidence that food security and economic development in Africa can be addressed more effectively with modern bioenergy than without it. This review is relevant to economic development, and in particular rural development, in African countries and poor countries elsewhere. Our findings are significant because they point to opportunities for development that are not fully realized, and because they highlight potential positive outcomes in domains wherein the impact of bioenergy has often been assumed to be negative.

## Abbreviations

BTU: British thermal unit
C: carbon
CAADP: Comprehensive Africa Agriculture Development Programme (framework)
CO_2_: carbon dioxide
E25: blend of 25% ethanol and 75% gasoline
EJ: exajoule
EU: European Union
FAO: Food and Agriculture Organization (United Nations)
gal: gallon
GDP: gross domestic product
GHG: greenhouse gas
GHI: Global Hunger Index
GW: gigawatt
ha: hectare
kW: kilowatt
kWh: kilowatt-hour
L: liter
Mha: million hectare
MDGs: Millenium Development Goals
Mtoe: megaton oil equivalent
MW: megawatt
MWe: megawatts of electricity
N: nitrogen
NEPAD: New Partnership for Africa’s Development
NGO: non-governmental organization
PIDA: Program for Infrastructure Development in Africa
TWh: terawatt-hour
v/v: volume per volume
UN: United Nations
US: United States
